# Ongoing Secondary Degeneration of the Limbic System in Patients With Ischemic Stroke: A Longitudinal MRI Study

**DOI:** 10.3389/fneur.2019.00154

**Published:** 2019-03-05

**Authors:** Muhammad E. Haque, Refaat E. Gabr, Khader M. Hasan, Sarah George, Octavio D. Arevalo, Alicia Zha, Susan Alderman, Jerome Jeevarajan, Manual F. Mas, Xu Zhang, Nikunj Satani, Elliott R. Friedman, Clark W. Sitton, Sean Savitz

**Affiliations:** ^1^Institute for Stroke and Cerebrovascular Diseases, University of Texas Health Science Center at Houston, Houston, TX, United States; ^2^Diagnostic and Interventional Imaging, University of Texas Health Science Center at Houston, Houston, TX, United States; ^3^TIRR Memorial Hermann Rehabilitation and Research, Houston, TX, United States; ^4^Biostatistics/Epidemiology/Research Design Component, Center for Clinical and Translational Sciences, McGovern Medical School at University of Texas Health Science Center at Houston (UTHealth), Houston, TX, United States

**Keywords:** ischemic stroke, limbic system atrophy, secondary degeneration, chronic loss of gray matter, longitudinal neuroimaging study

## Abstract

**Purpose:** Ongoing post-stroke structural degeneration and neuronal loss preceding neuropsychological symptoms such as cognitive decline and depression are poorly understood. Various substructures of the limbic system have been linked to cognitive impairment. In this longitudinal study, we investigated the post-stroke macro- and micro-structural integrity of the limbic system using structural and diffusion tensor magnetic resonance imaging.

**Materials and Methods:** Nineteen ischemic stroke patients (11 men, 8 women, average age 53.4 ± 12.3, range 18–75 years), with lesions remote from the limbic system, were serially imaged three times over 1 year. Structural and diffusion-tensor images (DTI) were obtained on a 3.0 T MRI system. The cortical thickness, subcortical volume, mean diffusivity (MD), and fractional anisotropy (FA) were measured in eight different regions of the limbic system. The National Institutes of Health Stroke Scale (NIHSS) was used for clinical assessment. A mixed model for multiple factors was used for statistical analysis, and *p*-values <0.05 was considered significant.

**Results:** All patients demonstrated improved NIHSS values over time. The ipsilesional subcortical volumes of the thalamus, hippocampus, and amygdala significantly decreased (*p* < 0.05) and MD significantly increased (*p* < 0.05). The ipsilesional cortical thickness of the entorhinal and perirhinal cortices was significantly smaller than the contralesional hemisphere at 12 months (*p* < 0.05). The cortical thickness of the cingulate gyrus at 12 months was significantly decreased at the caudal and isthmus regions as compared to the 1 month assessment (*p* < 0.05). The cingulum fibers had elevated MD at the ipsilesional caudal-anterior and posterior regions compared to the corresponding contralesional regions.

**Conclusion:** Despite the decreasing NIHSS scores, we found ongoing unilateral neuronal loss/secondary degeneration in the limbic system, irrespective of the lesion location. These results suggest a possible anatomical basis for post stroke psychiatric complications.

## Introduction

Stroke research and clinical management have been predominantly focused on the acute phase of injury and little is known about the rates of ongoing chronic degeneration. Despite clinical improvement, stroke survivors often suffer from persistent hemiparesis, cognitive decline, depression, and anxiety. A number of neuropsychiatric studies have shown an association between ischemic injury and cognitive impairment, progressive brain atrophy, impaired neuronal connectivity, compromised hemodynamics, and secondary neuronal degeneration (1–8).

Chronic thalamic neuronal loss remote from the lesion was noted in an animal study (9). Later research validated this evidence, and further found that neuroinflammation was not limited to the lesion area, but globally impacted the ipsilesional hemisphere (10). Another longitudinal primate study demonstrated ipsilesional hemispheric atrophy and secondary neuronal degeneration in the lentiform nucleus (11). Nishio et al. (12) demonstrated association between ischemic injury and secondary hippocampal degeneration (12). Another study using a rodent unilateral common carotid occlusion model reported a correlation between hippocampal atrophy and vascular cognitive impairment (13). Furthermore, several cross-sectional studies and a handful of longitudinal studies suggested an association between post-stroke tissue loss in the limbic system and cognitive impairment (14–22). However, these studies reported only two or three substructures of the limbic system and in some cases the primary lesions were within the limbic system.

The limbic system, composed of interconnected gray and white matter structures, plays a vital role in mood, emotion and cognitive functions such as memory and learning (23, 24). Gray matter structures include the amygdala, hippocampus, thalamus, parahippocampus, entorhinal cortex, hypothalamic nuclei, mammillary bodies, and cingulate gyrus. The primary white matter structures include the fornix and the cingulum bundle. Hippocampal, parahippocampal, and entorhinal atrophy has been associated with impairment of verbal memory in Alzheimer's disease and was used as surrogate markers for disease progression (25, 26).

In a recent serial study, Bivard et al. (27) reported an association between global gray matter atrophy and cognitive decline in patients with transient ischemic stroke (27). Post-stroke neuronal degeneration in brain regions remote from the primary infarct may underlie subcortical atrophy and cognitive impairment (7, 28–31). In this prospective longitudinal neuroimaging study, we sought to investigate ongoing secondary degeneration in eight substructures of the limbic system remote from the lesion using structural and diffusion tensor imaging (DTI) MRI to identify the most vulnerable substructures of the limbic system.

## Materials and Methods

### Patient Enrollment and Clinical Assessment

This study was approved by our Institutional Review Board and written informed consent was obtained prior to enrollment. The study enrolled 19 patients (10 male and 9 female, age range, 18–75 years) with unilateral ischemic stroke. At the time of enrollment, the patients had lesion size 0–100 cm^3^ and NIHSS ranges 1–25. Patients were excluded from the study if they were unable to undergo MRI examination, or if there was intracranial hemorrhage, cancer, seizure disorder, HIV, or pulmonary disease. Patients with lesions extending to any of the substructures within the limbic system were excluded from ipsi- and contralesional analysis.

The patients were imaged with a standard of care MRI protocol in the acute phase. Additional MRI exams were performed at 1 (1 M), 3 (3 M), and 12 (12 M) months of onset. The neurological assessment was done using the NIHSS at each follow-up visit. All patients underwent standard of care post-stroke rehabilitation.

### MRI Data Acquisition Protocol

All imaging experiments were performed on a whole-body 3.0 T Philips system (Philips Healthcare, Best, The Netherlands) using a multi-channel head coil. Structural MRI was obtained using 3D T1-weighted imaging (TE/TR = 3.66/8.2 ms, acquisition matrix = 256 × 256 × 170, FOV = 256 × 256 mm^2^). Anatomical localization of the lesion used Fluid-Attenuated Inversion Recovery (FLAIR) images (TE/TR/TI = 129 ms/4.8 s/1.6 s, acquisition matrix = 256 × 256 × 180, FOV = 256 × 256 mm^2^). DTI was used to assess microstructural integrity (TE/TR = 66 ms/7.1 s, acquisition matrix = 128 × 128, FOV = 256 × 256 mm^2^, slice thickness = 3 mm, no gaps; gradient scheme = 21 icosahedral, diffusion b-factor = 1,000 s/mm^2^) (32).

### Lesion Volume Measurements

A semi-automated algorithm in Analyze 12.0 (Analyze Direct Inc., KS, USA) was used to delineate lesion volume on FLAIR images by two raters. The rater selected two seed points within the hyper- and hypointense regions within the lesions and a region-growing algorithm automatically expanded the seed points into the 3D space of the image. Manual editing of the lesion volume was done when necessary.

### Subcortical Volume and Cortical Thickness Analysis

Cortical thickness and subcortical volumes of the limbic structures were measured by automatic parcellation and segmentation of T1w images using FreeSurfer v5.3.0 (http://surfer.nmr.mgh.harvard.edu/) with default processing settings (33). Reconstructed segmented volumes and cortical thickness were visually inspected and manually edited, when necessary. All measurements were made in both the ipsi- and contralesional hemispheres. Contralesional measurements were used as control.

### Substructure Measurements of the Limbic System

Changes in volume, cortical thickness, mean diffusivity (MD), and fractional anisotropy (FA) were measured in eight substructures of the limbic system in each hemisphere. The volumetric measurements included the amygdala, hippocampus, and thalamus. Cortical thickness was measured in the entorhinal cortex, perirhinal cortex, parahippocampal, and cingulate cortex. The MD and FA were measured in the amygdala, hippocampus, thalamus, and cingulum. The cingulate cortex and cingulum measurements were divided into four segments: rostral-anterior, caudal-anterior, posterior, and isthmus cingulate. Two neuroradiologists with a combined 22 years of experience identified the lesion locations. The volumetric measurements were normalized to the intracranial volume.

### Mean Diffusivity and Fractional Anisotropy Measurements

DTI preprocessing included volume realignment, non-brain tissue removal, and eddy current correction using FSL (http://www.fmrib.ox.ac.uk/fsl/). The diffusion tensor was calculated voxel-wise using multivariate linear fitting using DtiStudio (www.DtiStudio.org), and MD and FA maps were generated. The T1w images, lesion mask, and b0 images (non-gradient DTI images) were registered using rigid-body transformation in SPM12 (https://www.fil.ion.ucl.ac.uk/spm/software/spm12/).

MD and FA were measured by drawing a region-of-interest (ROI) containing ~25–30 voxels in gray matter (GM, thalamus, hippocampus, and amygdala) and white matter regions of the limbic system (WM, anterior, posterior, rostral, and caudal regions of the cingulum). The ROIs were placed in each region using the DTI color maps as a guide. Special care was given to ROI placement to ensure sampling of the same location at all the time points. The ROIs were overlaid on MD and FA maps to record the values.

### Statistical Analysis

Mixed model analysis evaluated effects of multiple factors and determined the correlation in repeated measurements (34). The fixed effects in the mixed model included stroke laterality (ipsilesional and contralesional), serial visits, and interactions between laterality, visits, and lesion volume. The random effects included the patient and interactions between patients and laterality. The specified random effects led to a nested covariance matrix and accounted for correlation of measurements of the same patient and different level of correlation for measurements on the same side of brain. Tests were two tailed and *p*-values <0.05 were considered significant. Statistical analyses were performed using the SAS software (version 9.4, the SAS Institute, Cary, NC).

## Results

The patients had a median NIHSS of 6 (range 1–25). All patients completed the 1 month MRI. One patient missed the 3 month MRI and three patients missed 12 month MRI. The lesions were identified as middle cerebral artery infarct (*n* = 10), posterior cerebral artery infarcts (*n* = 5), anterior cerebral artery infarct (*n* = 2), and pontine infarct (*n* = 2). The average lesion size was 29.44 ± 25.4 mm^3^ with inter-rater reliability of 97% at 1 month. Eleven patients had left hemispheric stroke and eight had right hemispheric stroke. The serial lesion volume, location, and severity of individual patients are listed in Table 1.

**Table 1:** Patient demographics.

**PID**	**Laterality**	**Lesion location**	**NIHSS**			**Lesion volume (mL)**				**Figure 1**
			**Acute**	**01M**	**03M**	**12M**	**01M**	**03M**	**12M**	
P01	Left	Genu and body of CC, SFG, CR, PreM	5	0	0	0	15.06	14.82	10.72	1f
P02	Left	STG, SMG, FG, OC, wernicke	4	4	4	2	61.47	78.80	79.89	1d
P03	Right	LPC, SMG, insula, TL, PRF	13	NA	5	3	55.22	35.46	39.73	1m
P04	Left	Insula, broca, RF, LN	20	NA	0	0	22.16	22.06	25.50	1g
P05	Left	FG, OL, VP	5	3	1	1	15.37	11.75	12.33	1i
P06	Right	Caudate and basal ganglia, parahippocampal	8	2	4	1	13.47	11.14	7.72	1h
P07	Left	Frontal lobe, precentral gyrus, operculum	2	1	0	–	18.14	8.31	–	1q
P08	Left	LN, caudate, putamen	7	2	0	–	16.56	19.13	–	1n
P09	Left	Cerebellum	1	0	–	0	32.57	NA	22.13	1a
P10	Right	Thalamic, TL, hippo, parahippocampal	6	3	2	2	24.75	16.90	13.52	1s
P11	Left	Thalamic, TL, hippo, parahippocampal	7	3	2	1	43.60	26.77	28.18	1r
P12	Left	FG, TL, BG, broca	25	2	2	3	34.75	32.27	43.68	1j
P13	Right	Pontine	6	2	1	0	0.37	0.34	0.37	1c
P14	Right	Occipital lobe	2	1	1	0	6.64	2.65	1.50	1l
P15	Right	Basal ganglia and motor cortex	14	2	0	–	30.06	15.39	–	1k
P16	Left	Insula, basal ganglia	20	15	9	8	77.60	60.80	55.57	1b
P17	Left	Operculum and caudate	20	5	4	4	87.19	61.95	66.83	1e
P18	Center^*^	Pontine	1	1	2	1	0.92	1.61	1.54	1o
P19	Right	Internal capsule	5	1	0	1	3.38	4.82	5.12	1p

CC, corpus callosum; SFG, superior frontal gyrus; CR, corona radiata; PreM, pre motor area; STG, superior temporal gyrus; SMG, superior marginal gyrus; FG, fusiform gyrus; OL, occipital lobe; LPC, lateral prefrontal cortex; TL, temporal lobe; PRE, peri-rolandic fissure; RF, Rolandic fissure; LN, lentiform nucleus; VP, visual pathway; BG, basal ganglia; NIHSS, National Institute of Health Stroke Scale. NA, no data obtained; –, missed follow-up visit;

* *lesion center to right (right hemisphere as ipsilesional)*.

**Figure 1:**
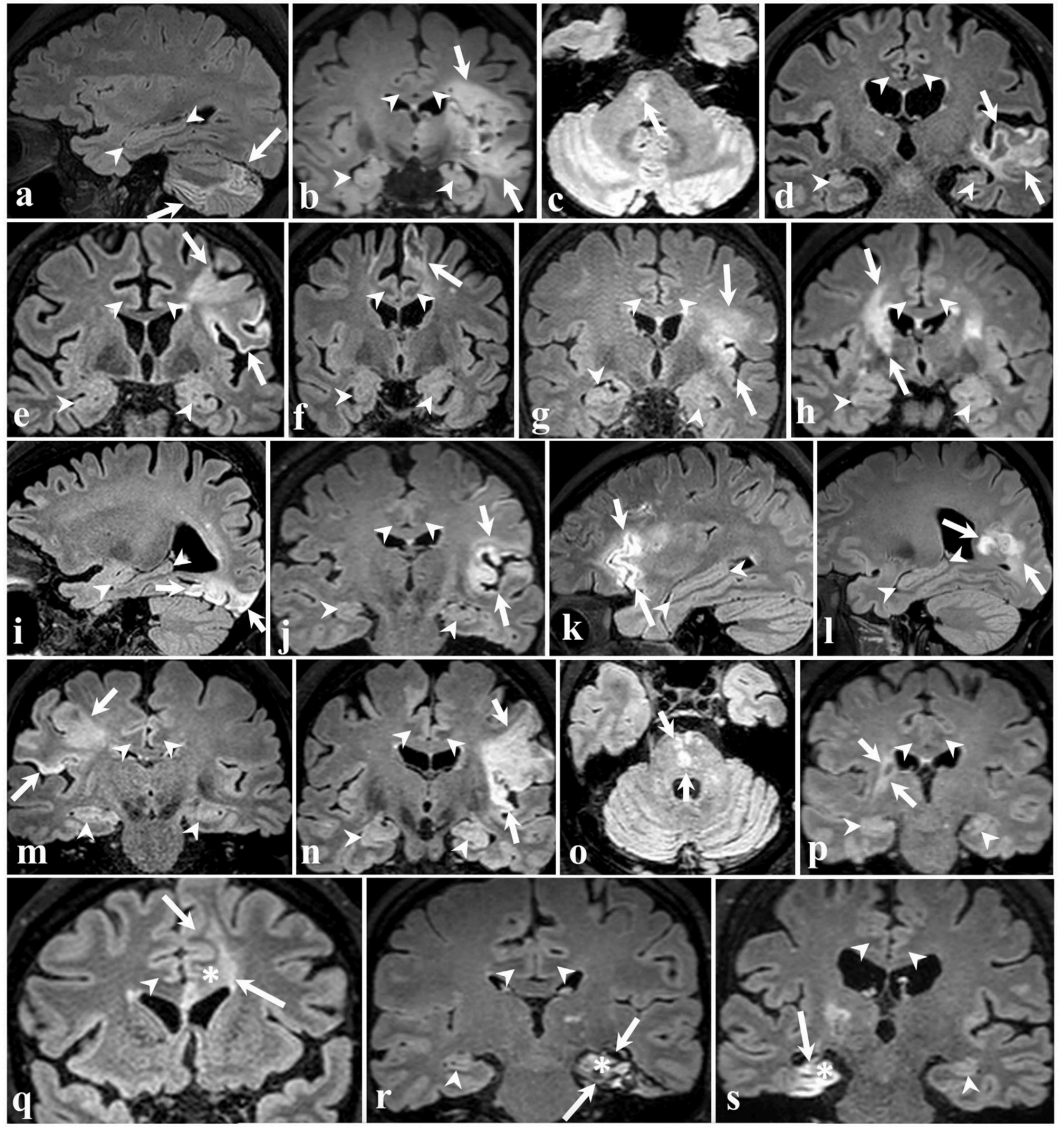
3D FLAIR brain images of all 19 participants in different planes. The arrows point to ischemic lesions and the arrowheads demarcate some of the limbic structures. In 16 patients **(a–p)** shown in the four top rows, the stroke did not affect limbic structures. The bottom row shows three cases wherein ischemia injury involves the left cingulate gyrus **(q)**, left hippocampus **(r)**, and right hippocampus **(s)**, marked by an asterisk.

**Figure 2:**
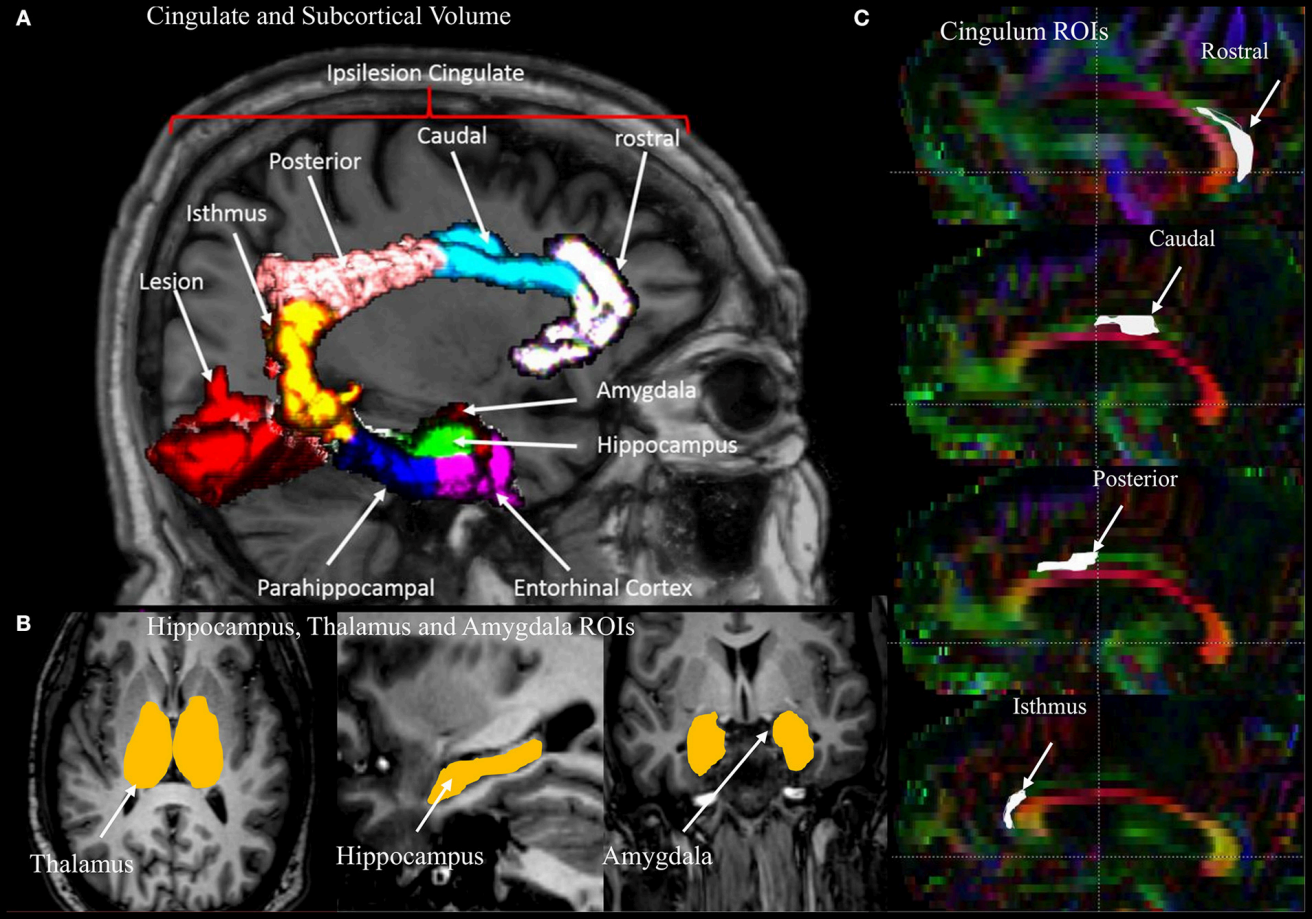
An illustration of the regions of interest (ROIs), segmented volumes, and regional parcellation of the limbic system used for measurements. **(A)** Four segments of the cingulate gyrus (rostral, caudal, posterior, isthmus) and two regions of the medial temporal lobe (entorhinal and parahippocampal cortecies) with lesion in red. **(B)** Segmented volumes of the thalamus, hippocampus, and amygdala. **(C)** Sagittal view of a DTI color map highlighting the four regions of the cingulum used for the measurements.

Representative MRI images depicting the approximate size and location of the lesions are shown in Figure 1. Most of the patients had lesions remote from the limbic system. Figure 2 is a graphic illustration of the segmented limbic system structures on T1w MRI and the ROI locations for the DTI measurement in one patient.

### Subcortical Volumes

**Figure 3:**
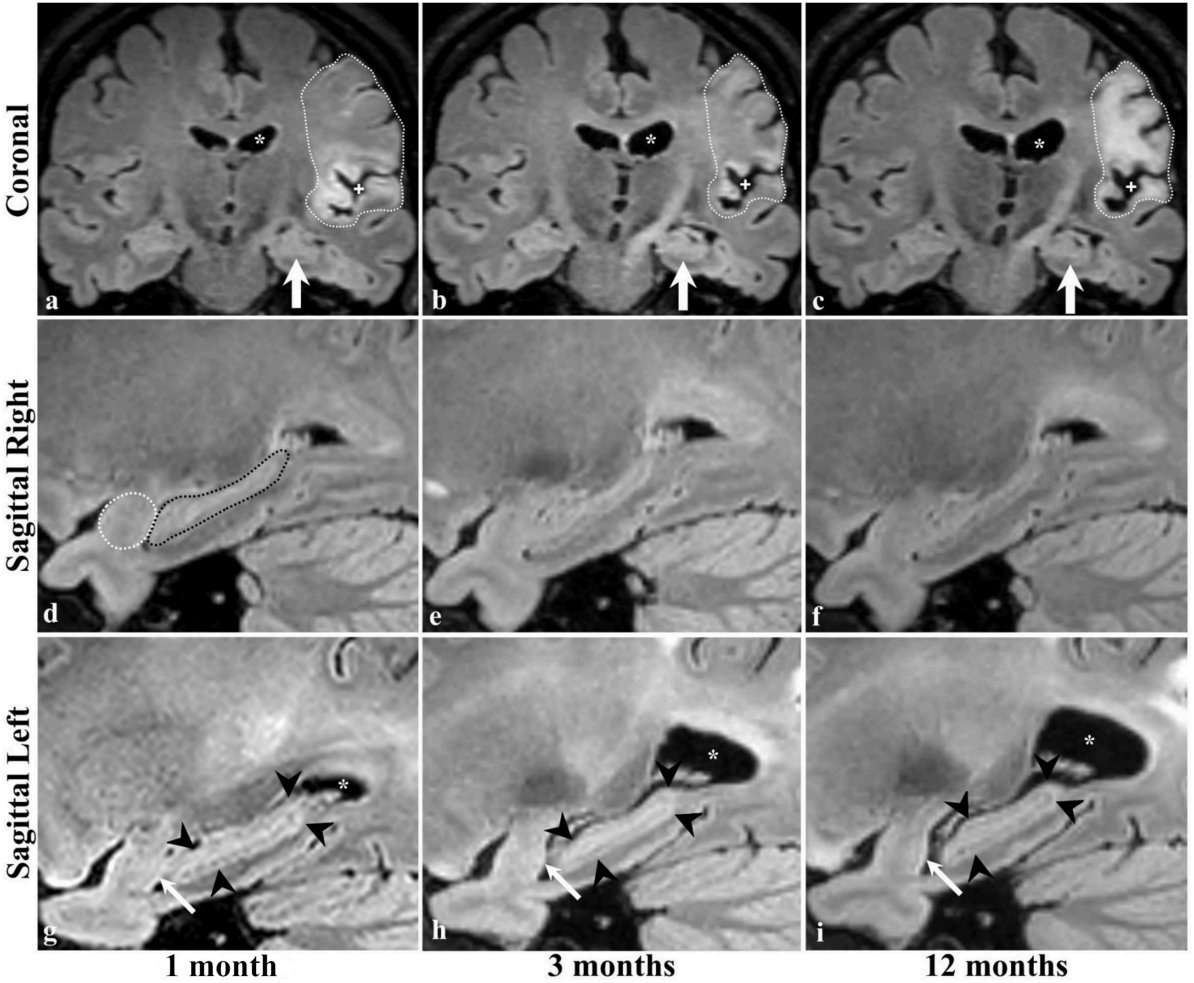
Brain MRI of a patient with ischemic stroke in the left MCA territory. Coronal images are at the level of the interthalamic adhesion at 1, 3 and 12 months of stroke (**a–c**, respectively), with concurrent sagittal images centered at the right **(d–f)** and left **(g–i)** hippocampi. Dotted lines **(a–c)** delineate the infarcted brain tissue and its expected evolution in time, including the increase in the parenchymal signal intensity (gliosis), the *ex-vivo* dilatation of the corresponding lateral ventricle (^*^ in the top and bottom rows), and the widening of the adjacent subarachnoid space (+ in top row) indicative of parenchymal volume loss and scarring. The left hippocampus was not affected by the ischemic event (arrows in **a–c**). Dotted lines in the image of the right temporal lobe **(d)** delineate the normal appearing amygdala (white) and hippocampus (black) and no changes were identified at 3 **(e)** or 12 **(f)** months. The left amygdala and hippocampus are normal in size and morphology at baseline examination **(g)**; however, there is progressive hippocampal atrophy in the follow-up scans (arrowheads in **g–i**). Interval volume loss of the left temporal amygdala can be identified by gradual straightening of its posterior contour (arrows in **g–i**).

**Figure 4:**
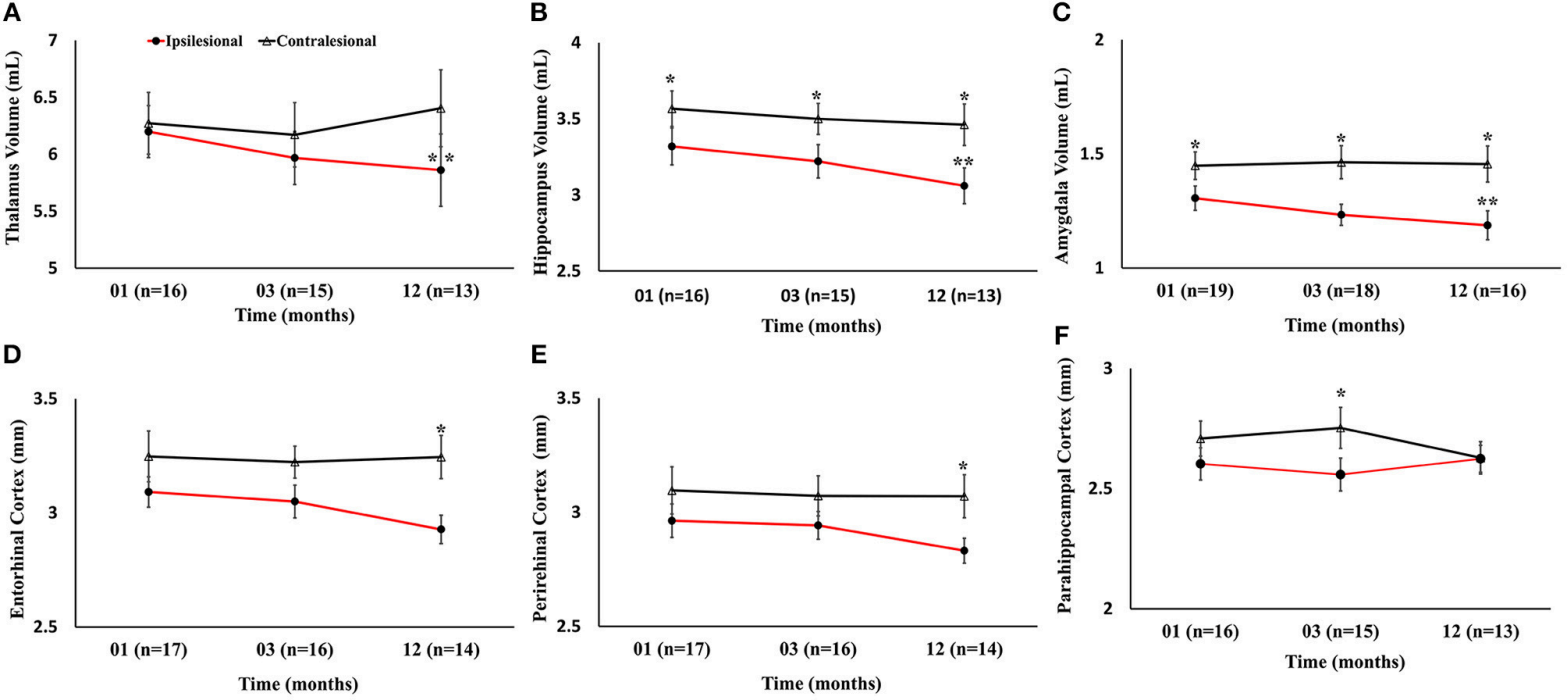
Serial volumetric and cortical thickness measurements in six gray matter regions of the limbic system. **(A–C)** Temporal decrease in ipsilesional volumes of the thalamus, hippocampus, and amygdala, compared to the contralesional side. **(D–F)** Changes in the ipsilesional cortical thickness of the entorhinal, perirhinal, and parahippocampal cortices. The asterisk (^*^) denotes significant (*p* < 0.05) hemispheric differences and the double asterisk (^**^) denotes significant (*p* < 0.05) temporal difference between first and last time points in the ipsilesional side. The error bars represent standard error in the mean.

**Figure 5:**
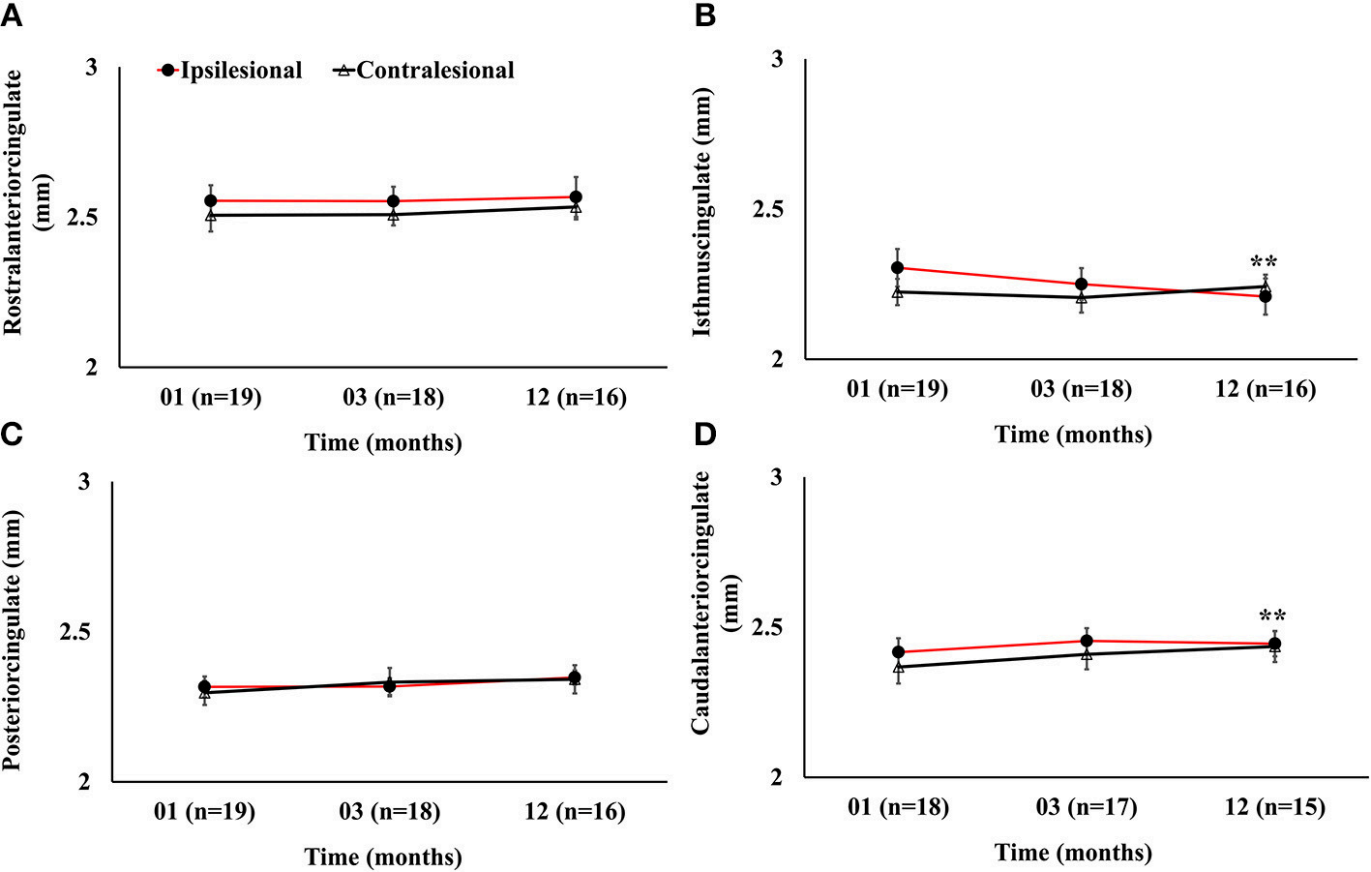
Serial cortical thickness measurements in four segments of the cingulate gyrus showing **(A)** no significant ipsilesional progressive change in the rostralanterior region of the cingulate; **(B)** significant progressive decrease in the isthmuscingulate; **(C)** no ipsilesional progressive or hemispheric change in the posteriorcingulate regions; and **(D)** ipsilesional progressive decrease in the caudalanteriorcingulate. The double asterisk symbol (^**^) denotes significant temporal ipsilesional difference between the first and last time point (*p* < 0.05). The error bars represent standard error in the mean.

Serial radiological assessment revealed remote structural atrophy in the ipsilesional hippocampus and amygdala, as shown for a representative patient in Figure 3. This was validated by quantitative volumetric analysis which demonstrated a significant reduction in the ipsilesional amygdala and hippocampus volume at 12 months (*p* < 0.05). The temporal ipsilesional thalamus volume was also significantly decreased (*p* < 0.05). The hemispheric comparison between ipsi and contralesional sides revealed significant reduction in the hippocampal and amygdala volumes but not the thalamic volume. The ipsilesional cortical thickness of the two sub-regions of the medial temporal lobe, entorhinal, and perirhinal cortices, was significantly reduced at 12 months compared to the contralesional side (*p* < 0.05), with the exception of the parahippocampal region (*p* = 0.10). There was no temporal decrease in the three ipsilesional medial temporal regions. The serial changes in the ipsilesional and contralesional volumes and cortical thickness are illustrated in Figure 4. The cortical thickness of the ipsilesional cingulate cortices was progressively decreased in the caudalanterior and isthmus regions (*p* < 0.05). The cortical thickness of the posteriorcingulate and rostralanterior regions showed longitudinal change, but no hemispheric differences were observed. The serial changes in the ipsi- and contralesional cingulate are shown in Figure 5.

### Diffusion Tensor Analysis

#### Gray Matter Fractional Anisotropy and Mean Diffusivity

**Figure 6:**
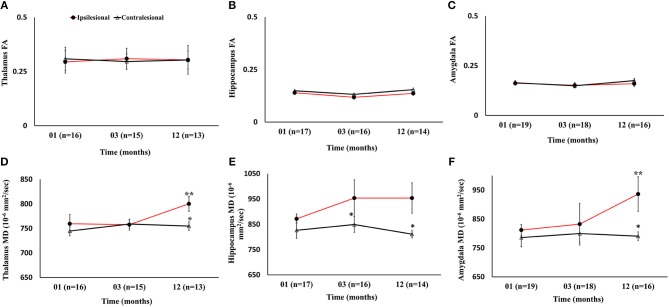
Serial diffusion tensor measurements in three gray matter regions of the limbic system. **(A–C)** Temporal changes in fractional anisotropy (FA) between the ipsilesional and contralesional thalamus, hippocampus, and amygdala. **(D–F)** Changes in the ipsilesional mean diffusivity (MD) in the thalamus, hippocampus, and amygdala. The asterisk (^*^) denotes significant hemispheric differences (*p* < 0.05) and the double asterisk (^**^) denotes significant temporal ipsilesional difference between the first and last time point (*p* < 0.05). The error bars represent standard error in the mean.

There were no progressive changes in ipsilesional FA and no differences between the ipsi- and contralesional thalamus, hippocampus, and amygdala. A significant increase in the MD of the ipsilesional thalamus, and amygdala was found at 12 months compared to 1 month (*p* < 0.05). The comparison between ipsi- and contralesional MD of these regions showed significantly higher values in the ipsilesional hemisphere (*p* < 0.05) at 12 months. The DTI findings in subcortical gray matter are summarized in Figure 6.

#### White Matter Fractional Anisotropy and Mean Diffusivity

**Figure 7:**
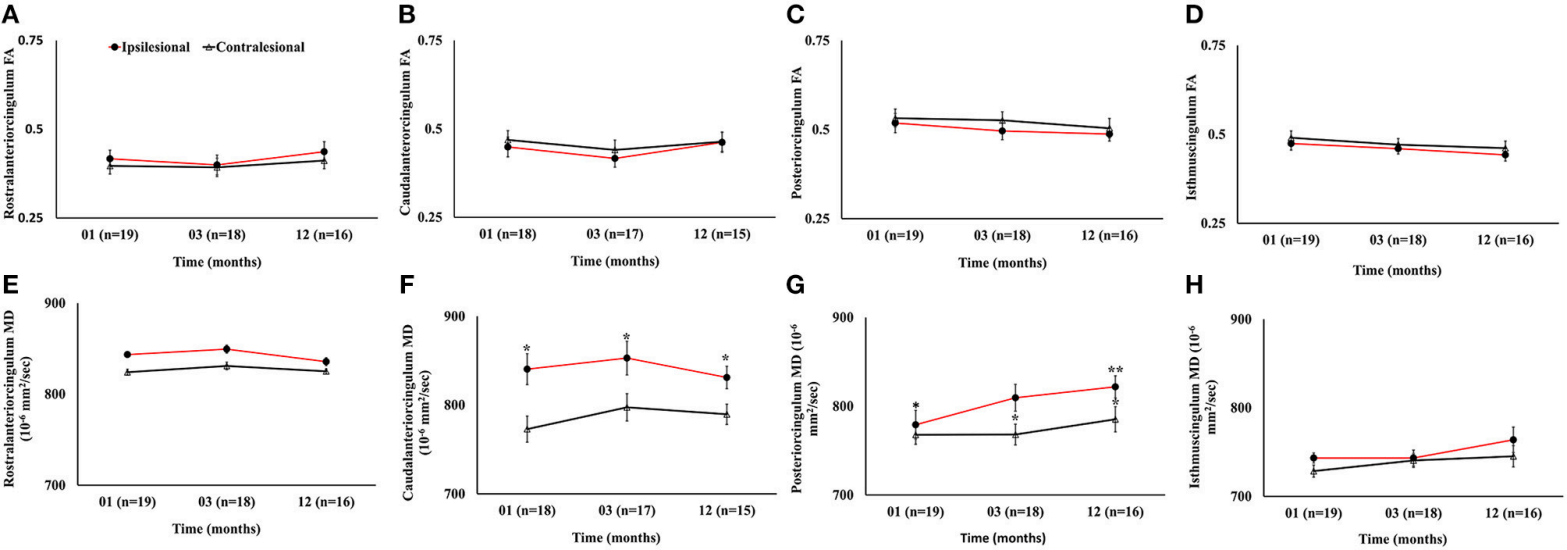
Serial diffusion tensor measurements in four white matter regions of cingulum fibers in the limbic system. **(A–D)** Temporal changes in fractional anisotropy (FA) between the ipsilesional and contralesional rostral, caudal, posterior, and isthmus regions of the cingulum. **(E–H)** Temporal changes in the ipsilesional and contralesional mean diffusivity (MD) of the rostral, caudal, posterior, and isthmus. The asterisk (^*^) denotes significant hemispheric differences (*p* < 0.05) and the double asterisk (^**^) denotes significant temporal ipsilesional difference between the first and last time point (*p* < 0.05). The error bars represent standard error in the mean.

The ipsilesional caudal and posterior cingulum fibers showed significant increase in MD as compared to the contralesional side (*p* < 0.05). However, only the ipsilesional posteriorcingulum fiber demonstrated elevated MD between 1 and 12 months (*p* < 0.05). There were no progressive or hemispheric differences in MD of the rostral and isthmus regions of the cingulum. Unlike MD, there was no statistically significant difference between ipsi- and contralesional FA in any segment. The overall changes in FA and MD (Figure 7) and volumetric, cortical thickness, and microstructural changes are summarized in Table 2.

**Table 2:** A summary of results comparing progressive changes in the ipsilesional measurement between 1 and 12 months (progressive), and between the ipsilesional and the contralesional hemispheres (Hemispheric).

**Structures**	**Measurements**	**Progressive**^**†**^	**Hemispheric**^**††**^
Amygdala	Volume	↓	↓
	MD	↑	↑
	FA	—	—
Hippocampus	Volume	↓	↓
	MD	—	↑
	FA	—	—
Thalamus	Volume	↓	—
	MD	↑	↑
	FA	—	—
Entorhinal cortex	Cortical thickness	—	↓
Perirhinal cortex	Cortical thickness	—	↓
Parahippocampal	Cortical thickness	—	↓
Caudalanteriorcingulate	Cortical thickness	↓	—
Isthmuscingulate	Cortical thickness	↓	—
Posteriorcingulate	Cortical thickness	—	—
Rostralanteriorcingulate	Cortical thickness	—	—
Caudalanterior cingulum	MD	—	↑
	FA	—	—
Isthamus cingulum	MD	—	—
	FA	—	—
Posterior cingulum	MD	↑	↑
	FA	—	—
Rostralanterior cingulum	MD	—	—
	FA	—	—

† *Ipsilesional increase (↑) or decrease (↓) in measurements between 1 and 12M*.

†† *Comparisons between ipsi- and contralesional measurement side at any visit. ↑ and ↓ are statistically significant P < 0.05. — No statistically significant change*.

The correlation between lesion volume and changes in subcortical volumes, cortical thickness, FA, and MD showed no effect except for MD of the ipsilesional amygdala, which correlated significantly with lesion volume (*p* < 0.05).

## Discussion

To the best of our knowledge, this is the first longitudinal study to combine volumetry, cortical thickness, and DTI measurements to track post-stroke regional macro- and microstructural changes in the limbic system. We found the rate of progressive degeneration, when compared to the contralesional side, to be heterogeneous. While none of the patients in this study had a subcortical infarct, alterations in the subcortical structures of the limbic system were observed. We identified ipsilesional thalamus, hippocampus, amygdala, entorhinal, and perirhinal cortices to be most vulnerable to degeneration. Unlike the thalamus, the hippocampal and amygdala volumes were significantly reduced within 1 month of onset, suggesting degeneration onset within the acute to subacute period. This was further supported by increased MD of the ipsilesional hippocampus and amygdala at the sub-chronic phase, which occurred later in the thalamus during the chronic phase. Our data show no correlation between lesion volume and neuronal degeneration, with the exception of increased MD in the amygdala.

In animal models, positron emission tomography studies have shown secondary degeneration as a result of neuronal loss in stroke (10). Our data suggest that neuronal loss in the ipsilesional amygdala and hippocampus precedes that in the thalamus. Hippocampal volume loss has been recognized as a hallmark of cognitive impairment in Alzheimer's disease, and was recently suggested to play a role in stroke as well (21, 26). Post-stroke hippocampal atrophy is thought to be a predictor for post-stroke cognitive impairment (35). In a recent study, entorhinal atrophy and hippocampal structural deformation, but not hippocampal volume, were strongly associated with post-stroke cognitive impairment (36). We also found ipsilesional perirhinal atrophy, in addition to the entorhinal atrophy.

In our study, the observed tissues volume observed was accompanied by microstructural changes on DTI-derived scalar measures. The significantly increased MD in the ipsilesional thalamus, hippocampus, and amygdala suggests changes in water mobility due to early cellular breakdown. These findings are in agreement with previous post stroke imaging studies (37–40) and supports its potential role as a surrogate marker of poststroke unilateral neuronal loss. These microstructural changes could be secondary to Wallerian degeneration of the white matter pathways of the limbic system, which are connected to the brain and cerebellum. The other mechanism could be cytotoxic, vasogenic edema following the primary injury to fiber tracts.

Global neuroinflammation suggested in animal stroke models could be a contributing factor to remote neuronal loss. Furthermore, absence of temporal changes in structural and microstructural measures in the contralesional hemisphere suggests that structural disintegration is confined to the ipsilesional hemisphere. Future studies with a larger number of participants are needed to determine the existence and clinical significance of unilateral progressive volume loss, cortical thinning, and increased diffusivity, and to determine their contributions to cognitive impairment.

Our results show significant, progressive ipsilesional cortical thinning in the caudalanterior and isthmus regions of the cingulate cortex, compared to previously reported significant thinning in the posterior regions of cingulate (20). This may be due to differences in measurement methods. We used FreeSurfer analysis for average thickness, while the previous study used voxel-based morphometry (20).

Furthermore, our results show significant elevated ipsilesional MD in the caudal and posterior segments of the cingulum but not in the rostral and isthmus regions, suggesting selective white matter vulnerability. Unexpectedly, we did not observe changes in ipsilesional FA in the caudal and posterior regions, suggesting that changes in MD may precede those in FA.

The limitations of our study include the small sample size and heterogeneity in lesion volume and location between patients. The study duration of 12 months may be too short to track long-term tissue loss. Lack of serial neurophysiological measurements prevents ascribing causality of these structural changes to post-stroke neuropsychological impairment. Cognitive assessment and neurological functional will be collected in future studies to determine the clinical relevance of our imaging findings.

In conclusion, this study uncovered ongoing micro- and macrostructural changes in the limbic system long after injury, even with clinical improvement assessed with NIHSS scores. These findings provide insight into the mechanisms leading to cognitive and psychiatric impairments often observed after stroke. Further, we showed that many of these effects exist even when the injury site is remote from the limbic structures. Nonetheless, these ongoing and non-localized complications of stroke demand further studies to better understand their mechanisms and develop appropriate interventions.

## Human Subject Participants

This Study was approved by the McGovern Medical School, University of Texas Health Sciences Center at Houston Committee for the Protection of Human Subjects and by the Memorial Hermann Office of Research.

## Author Contributions

MH designed the study, carried MRI quantitative analysis, and drafted the manuscript. RG contributed to data analysis, assisted in optimizing MRI acquisition. KH provided qualitative and quantitative quality assurance of images and data analysis. SG and SA recruited, consented, and obtained patient's serial neurological assessment. OA and AZ performed region of interest analysis. JJ provided free surfer analysis. MM assisted in patient's recruitment from his facility. NS assisted in creating plots and figures. XZ performed statistical analysis. EF and CS provided radiology reports and assisted in lesion locations. SS supervised the study and provided necessary resources. All authors contributed to and approved the final manuscript.

### Conflict of Interest Statement

The authors declare that the research was conducted in the absence of any commercial or financial relationships that could be construed as a potential conflict of interest.

## References

[1] Sachdev PS Chen X Joscelyne A Wen W Brodaty H. Amygdala in stroke/transient ischemic attack patients and its relationship to cognitive impairment and psychopathology: the Sydney Stroke Study. Am J Geriatr Psychiatry. (2007) 15:487–96. 10.1097/JGP.0b013e3180581fe6 17545449

[2] Jack CR Jr Weigand SD Shiung MM Przybelski SA O'brien PC Gunter JL. Atrophy rates accelerate in amnestic mild cognitive impairment. Neurology. (2008) 70:1740–52. 10.1212/01.wnl.0000281688.77598.35 18032747 PMC2734477

[3] Stebbins GT Nyenhuis DL Wang C Cox JL Freels S Bangen K. Gray matter atrophy in patients with ischemic stroke with cognitive impairment. Stroke. (2008) 39:785–93. 10.1161/STROKEAHA.107.507392 18258824

[4] Lee SR Choi B Paul S Seo JH Back DB Han JS. Depressive-like behaviors in a rat model of chronic cerebral hypoperfusion. Transl Stroke Res. (2015) 6:207–14. 10.1007/s12975-014-0385-3 25541087

[5] Yassi N Malpas CB Campbell BC Moffat B Steward C Parsons MW. Contralesional thalamic surface atrophy and functional disconnection 3 months after ischemic stroke. Cerebrovasc Dis. (2015) 39:232–41. 10.1159/000381105 25823493

[6] Bustamante A Garcia-Berrocoso T Rodriguez N Llombart V Ribo M Molina C. Ischemic stroke outcome: a review of the influence of post-stroke complications within the different scenarios of stroke care. Eur J Intern Med. (2016) 29:9–21. 10.1016/j.ejim.2015.11.030 26723523

[7] Diao Q Liu J Wang C Cheng J Han T Zhang X. Regional structural impairments outside lesions are associated with verbal short-term memory deficits in chronic subcortical stroke. Oncotarget. (2017) 8:30900–7. 10.18632/oncotarget.15882 28427203 PMC5458176

[8] Yu X Yang L Song R Jiaerken Y Yang J Lou M. Changes in structure and perfusion of grey matter tissues during recovery from Ischaemic subcortical stroke: a longitudinal MRI study. Eur J Neurosci. (2017) 46:2308–14. 10.1111/ejn.13669 28833690

[9] Justicia C Ramos-Cabrer P Hoehn M. MRI detection of secondary damage after stroke: chronic iron accumulation in the thalamus of the rat brain. Stroke. (2008) 39:1541–7. 10.1161/STROKEAHA.107.503565 18323485

[10] Walberer M Jantzen SU Backes H Rueger MA Keuters MH Neumaier B. *In-vivo* detection of inflammation and neurodegeneration in the chronic phase after permanent embolic stroke in rats. Brain Res. (2014) 1581:80–8. 10.1016/j.brainres.2014.05.030 24905627

[11] Bihel E Pro-Sistiaga P Letourneur A Toutain J Saulnier R Insausti R. Permanent or transient chronic ischemic stroke in the non-human primate: behavioral, neuroimaging, histological, and immunohistochemical investigations. J Cereb Blood Flow Metab. (2010) 30:273–85. 10.1038/jcbfm.2009.209 19794396 PMC2949113

[12] Nishio K Ihara M Yamasaki N Kalaria RN Maki T Fujita Y. A mouse model characterizing features of vascular dementia with hippocampal atrophy. Stroke. (2010) 41:1278–84. 10.1161/STROKEAHA.110.581686 20448204

[13] Zuloaga KL Zhang W Yeiser LA Stewart B Kukino A Nie X. Neurobehavioral and imaging correlates of hippocampal atrophy in a mouse model of vascular cognitive impairment. Transl Stroke Res. (2015) 6:390–8. 10.1007/s12975-015-0412-z 26040424 PMC4561019

[14] Delano-Wood L Stricker NH Sorg SF Nation DA Jak AJ Woods SP. Posterior cingulum white matter disruption and its associations with verbal memory and stroke risk in mild cognitive impairment. J Alzheimers Dis. (2012) 29:589–603. 10.3233/JAD-2012-102103 22466061 PMC3341099

[15] Gauthier LV Taub E Mark VW Barghi A Uswatte G. Atrophy of spared gray matter tissue predicts poorer motor recovery and rehabilitation response in chronic stroke. Stroke. (2012) 43:453–7. 10.1161/STROKEAHA.111.633255 22096036 PMC3265680

[16] Grysiewicz R Gorelick PB. Key neuroanatomical structures for post-stroke cognitive impairment. Curr Neurol Neurosci Rep. (2012) 12:703–8. 10.1007/s11910-012-0315-2 23070618

[17] Lopes MA Firbank MJ Widdrington M Blamire AM Kalaria RN O'brien JT. Post-stroke dementia: the contribution of thalamus and basal ganglia changes. Int Psychogeriatr. (2012) 24:568–76. 10.1017/S1041610211002195 22153202

[18] Dang C Liu G Xing S Xie C Peng K Li C. Longitudinal cortical volume changes correlate with motor recovery in patients after acute local subcortical infarction. Stroke. (2013) 44:2795–801. 10.1161/STROKEAHA.113.000971 23929747

[19] Duering M Righart R Wollenweber FA Zietemann V Gesierich B Dichgans M. Acute infarcts cause focal thinning in remote cortex via degeneration of connecting fiber tracts. Neurology. (2015) 84:1685–92. 10.1212/WNL.0000000000001502 25809303 PMC4409580

[20] Matsuoka K Yasuno F Taguchi A Yamamoto A Kajimoto K Kazui H. Delayed atrophy in posterior cingulate cortex and apathy after stroke. Int J Geriatr Psychiatry. (2015) 30:566–72. 10.1002/gps.4185 25092799

[21] Selnes P Grambaite R Rincon M Bjornerud A Gjerstad L Hessen E. Hippocampal complex atrophy in poststroke and mild cognitive impairment. J Cereb Blood Flow Metab. (2015) 35:1729–37. 10.1038/jcbfm.2015.110 26036934 PMC4635227

[22] Chenfei Y Jun W Xuhui C Changle Z Hengtong L Shuai M Structural changes of cingulate cortex in post stroke depression. Conf Proc IEEE Eng Med Biol Soc. (2016) 2016:1099–102. 10.1109/embc.2016.7590895 28268517

[23] Mark LP Daniels DL Naidich TP Borne JA. Limbic system anatomy: an overview. AJNR Am J Neuroradiol. (1993) 14:349–52. 8456710 PMC8332961

[24] Morgane PJ Galler JR Mokler DJ. A review of systems and networks of the limbic forebrain/limbic midbrain. Prog Neurobiol. (2005) 75:143–60. 10.1016/j.pneurobio.2005.01.001 15784304

[25] Apostolova LG Green AE Babakchanian S Hwang KS Chou YY Toga AW. Hippocampal atrophy and ventricular enlargement in normal aging, mild cognitive impairment (MCI), and Alzheimer Disease. Alzheimer Dis Assoc Disord. (2012) 26:17–27. 10.1097/WAD.0b013e3182163b62 22343374 PMC3286134

[26] Hashimoto M Araki Y Takashima Y Nogami K Uchino A Yuzuriha T. Hippocampal atrophy and memory dysfunction associated with physical inactivity in community-dwelling elderly subjects: the Sefuri study. Brain Behav. (2017) 7:e00620. 10.1002/brb3.620 28239530 PMC5318373

[27] Bivard A Lillicrap T Marechal B Garcia-Esperon C Holliday E Krishnamurthy V. Transient ischemic attack results in delayed brain atrophy and cognitive decline. Stroke. (2018) 49:384–90. 10.1161/STROKEAHA.117.019276 29301970

[28] Thomalla G Glauche V Koch MA Beaulieu C Weiller C Rother J. Diffusion tensor imaging detects early Wallerian degeneration of the pyramidal tract after ischemic stroke. Neuroimage. (2004) 22:1767–74. 10.1016/j.neuroimage.2004.03.041 15275932

[29] Ahdab R Kikano R Saade H Riachi N. Early corticospinal tract Wallerian degeneration versus mesencephalic substantia nigra degeneration secondary to striatal stroke. Clin Neurol Neurosurg. (2014) 118:101–2. 10.1016/j.clineuro.2013.12.005 24468328

[30] Seghier ML Ramsden S Lim L Leff AP Price CJ. Gradual lesion expansion and brain shrinkage years after stroke. Stroke. (2014) 45:877–9. 10.1161/STROKEAHA.113.003587 24425126

[31] Werden E Cumming T Li Q Bird L Veldsman M Pardoe HR. Structural MRI markers of brain aging early after ischemic stroke. Neurology. (2017) 89:116–24. 10.1212/WNL.0000000000004086 28600458 PMC5501937

[32] Hasan KM Narayana PA. Computation of the fractional anisotropy and mean diffusivity maps without tensor decoding and diagonalization: theoretical analysis and validation. Magn Reson Med. (2003) 50:589–98. 10.1002/mrm.10552 12939767

[33] Fischl B Sereno MI Dale AM Cortical surface-based analysis. II: inflation, flattening, and a surface-based coordinate system. Neuroimage. (1999) 9:195–207. 10.1006/nimg.1998.0396 9931269

[34] Littell RC Henry PR Ammerman CB. Statistical analysis of repeated measures data using SAS procedures. J Anim Sci. (1998) 76:1216–31. 10.2527/1998.7641216x 9581947

[35] Mehrabian S Raycheva M Petrova N Janyan A Petrova M Traykov L. Neuropsychological and neuroimaging markers in prediction of cognitive impairment after ischemic stroke: a prospective follow-up study. Neuropsychiatr Dis Treat. (2015) 11:2711–9. 10.2147/NDT.S86366 26527875 PMC4621206

[36] Delattre C Bournonville C Auger F Lopes R Delmaire C Henon H. Hippocampal deformations and entorhinal cortex atrophy as an anatomical signature of long-term cognitive impairment: from the MCAO rat model to the stroke patient. Transl Stroke Res. (2017) 9:294–305. 10.1007/s12975-017-0576-9 29034421

[37] Herve D Molko N Pappata S Buffon F Lebihan D Bousser MG. Longitudinal thalamic diffusion changes after middle cerebral artery infarcts. J Neurol Neurosurg Psychiatry. (2005) 76:200–5. 10.1136/jnnp.2004.041012 15654032 PMC1739509

[38] Schaapsmeerders P Van Uden IW Tuladhar AM Maaijwee NA Van Dijk EJ Rutten-Jacobs LC. Ipsilateral hippocampal atrophy is associated with long-term memory dysfunction after ischemic stroke in young adults. Hum Brain Mapp. (2015) 36:2432–42. 10.1002/hbm.22782 25757914 PMC6869088

[39] Kliper E Ben Assayag E Korczyn AD Auriel E Shopin L Hallevi H. Cognitive state following mild stroke: a matter of hippocampal mean diffusivity. Hippocampus. (2016) 26:161–9. 10.1002/hipo.22500 26222988

[40] Hosseini AA Meng D Simpson RJ Auer DP. Mesiotemporal atrophy and hippocampal diffusivity distinguish amnestic from non-amnestic vascular cognitive impairment. Eur J Neurol. (2017) 24:902–11. 10.1111/ene.13299 28547878 PMC5518192

